# Impact of congenital cytomegalovirus infection on transcriptomes from archived dried blood spots in relation to long-term clinical outcome

**DOI:** 10.1371/journal.pone.0200652

**Published:** 2018-07-19

**Authors:** Roberta Rovito, Hans-Jörg Warnatz, Szymon M. Kiełbasa, Hailiang Mei, Vyacheslav Amstislavskiy, Ramon Arens, Marie-Laure Yaspo, Hans Lehrach, Aloys C. M. Kroes, Jelle J. Goeman, Ann C. T. M. Vossen

**Affiliations:** 1 Department of Medical Microbiology, Leiden University Medical Center, Leiden, The Netherlands; 2 Otto Warburg Laboratory Gene Regulation and Systems Biology of Cancer, Max Planck Institute for Molecular Genetics, Berlin, Germany; 3 Department of Biomedical Data Sciences, Leiden University Medical Center, Leiden, The Netherlands; 4 Sequencing Analysis Support Core, Leiden University Medical Center, Leiden, The Netherlands; 5 Department of Immunohematology and Blood Transfusion, Leiden University Medical Center, Leiden, The Netherlands; 6 Alacris Theranostics GmbH, Berlin, Germany; University of St Andrews, UNITED KINGDOM

## Abstract

Congenital Cytomegalovirus infection (cCMV) is the leading infection in determining permanent long-term impairments (LTI), and its pathogenesis is largely unknown due to the complex interplay between viral, maternal, placental, and child factors. The cellular activity, considered to be the result of the response to exogenous and endogenous factors, is captured by the determination of gene expression profiles. In this study, we determined whole blood transcriptomes in relation to cCMV, CMV viral load and LTI development at 6 years of age by using RNA isolated from neonatal dried blood spots (DBS) stored at room temperature for 8 years. As DBS were assumed to mainly reflect the neonatal immune system, particular attention was given to the immune pathways using the global test. Additionally, differential expression of individual genes was performed using the voom/limma function packages. We demonstrated feasibility of RNA sequencing from archived neonatal DBS of children with cCMV, and non-infected controls, in relation to LTI and CMV viral load. Despite the lack of statistical power to detect individual genes differences, pathway analysis suggested the involvement of innate immune response with higher CMV viral loads, and of anti-inflammatory markers in infected children that did not develop LTI. Finally, the T cell exhaustion observed in infected neonates, in particular with higher viral load, did not correlate with LTI, therefore other mechanisms are likely to be involved in the long-term immune dysfunction. Despite these data demonstrate limitation in determining prognostic markers for LTI by means of transcriptome analysis, this exploratory study represents a first step in unraveling the pathogenesis of cCMV, and the aforementioned pathways certainly merit further evaluation.

## Introduction

Human Cytomegalovirus (CMV) is one of the most common causes of congenital viral infection, leading to a significant number of children with permanent disabilities. The overall birth prevalence of congenital CMV infection (cCMV) in industrialized countries is between 0.6% and 0.7% [1, 2]. Among the congenitally infected infants, 12.7% are estimated to have symptoms at birth, ranging from mild, such as petechiae, to severe, such as microcephaly [1, 2]. An estimated 40–58% of these symptomatic children develop permanent long-term disabilities, such as hearing loss, cognitive and motor developmental delay [1]. Although symptomatic neonates have a considerable risk to develop permanent long-term impairments (LTI), approximately 13% of the asymptomatic children will also develop permanent LTI [1]. Despite the current insights into the clinical outcome of cCMV, the multifactorial process that determines whether a child is symptomatic at birth or will develop LTI is largely unknown.

The control of cCMV, and cCMV-related disease, may be the result of a complex interaction between viral, maternal, placental, fetal and child factors [3]. The clinical impact of cCMV has mainly been evaluated in relation to maternal factors, such as the CMV immune status before pregnancy or the time of vertical transmission. The vertical transmission rate is higher among women without prior CMV infection than among previously exposed women [2], indicating that pre-existing immunity can be protective. Vertical transmission occurring in the first 20 weeks of pregnancy leads to a worse clinical outcome than transmission occurring later in pregnancy [4, 5]. The latter is probably related to an increased susceptibility to infection due to fetal organogenesis, and a still developing fetal immune system. Although the pathogenesis of LTI is poorly understood, the fetal and neonatal immune system likely play an important role in controlling the infection, thereby influencing LTI development [3]. Several studies have demonstrated a CMV-specific adaptive immune response in congenitally infected children, such as γδ and αβ T cells or B cells [6–10], as well as an innate immune response [11, 12]. However, only few studies have evaluated these responses in relation to clinical outcome at birth, whereas the majority has not done so in relation to LTI development. An increase of NK cells was observed in congenitally infected children, and their frequency was higher in those who were symptomatic at birth [11]. In proteomic studies, an increase of macrophage-derived cytokines was observed in congenitally infected children, whereas an increase of β-defensin was observed in those who were asymptomatic at birth [12]. Moreover, the cytokine profile of congenitally infected children, both asymptomatic and symptomatic, was different from that of their mothers with primary infection [13].

The gene expression profile captures a snapshot of the cellular activity which is the result of the response to genetic, environmental and epigenetic factors [14]. After having established, through forensic studies, that reliable RNAs can be extracted from dried stains, a considerable amount of studies focused on neonatal dried blood spots (DBS) because they represent an important archived, and readily accessible specimen to study factors of disease development. Indeed, DBS are usually collected at birth for the screening of rare genetic metabolic disorders, and are stored for several years [15]. Previous studies have shown that quantitative RNA measurements, either with microarrays or RNA-seq, can be performed on neonatal DBS stored at room temperature for up to 9 years [14, 16–18]. Additionally, since the transcriptional profiles of RNA derived from DBS in mice, stored for several months at room temperature, correlated with those from fresh whole blood [19], we assumed this may also be the case in humans. The transcriptome varies according to the cell types studied, and certain RNA markers are tissue-specific. Tissue-specific RNA molecules have been successfully extracted from blood and saliva stains, dried at room temperature for up to 16 years, and used for genome-wide expression analysis [20, 21]. Since DBS are produced by spotting whole blood on filter paper, they were assumed to mainly reflect the neonatal immune system.

The aim of this exploratory study was to evaluate the feasibility of transcriptome analysis from archived neonatal DBS in relation to cCMV and LTI development. In particular, we wanted to determine whether the neonatal immune system at birth may be a determinant of LTI development at 6 years of age. This would provide insights into the immune regulation of cCMV, and, by identifying prognostic markers for clinical outcome, could provide the means to introduce the long-debated newborn screening program for CMV in DBS by defining subgroups of infants that would benefit from clinical and audiological follow-up, and possibly antiviral treatment [22]. Our investigations revealed that transcriptome analysis of RNA from neonatal DBS stored at room temperature for 8 years of a nation-wide retrospective cohort of children with cCMV and controls is possible, and could potentially be used to unravel the pathogenesis of cCMV and CMV-related disease.

## Materials and methods

### Study population and clinical data

A previously described nationwide, retrospective cohort was used in this study [23]. The cohort was derived from a total group of 31,484 children, born in 2008 in the Netherlands, which was retrospectively tested for cCMV by PCR of CMV DNA in neonatal DBS at five years of age. In total, 156 children (0.5%) were diagnosed with cCMV. Clinical data were retrieved from 133 congenitally CMV-infected children and from 274 non-infected children. Children were defined as symptomatic at birth if they had one or more of the following signs or symptoms in the neonatal period: prematurity, being small for gestational age, microcephaly, hepato- or splenomegaly, generalized petechiae or purpura, hypotonia, abnormal laboratory findings (elevated liver transaminases, hyperbilirubinemia, neutropenia or thrombocytopenia), cerebral ultrasound abnormalities, ophthalmologic abnormalities or neonatal hearing impairment. LTI was defined as the presence of impairment in one or more domain (hearing, visual, neurological, motor, cognitive and speech-language). The cCMV associated LTI in the original cohort has been described in detail [24]. In brief, hearing impairment was defined as sensorineural hearing loss ≥ 40 dB; visual impairment was defined as a visual acuity below 0.3; neurological impairment included cerebral palsy, epilepsy, microcephaly, autism spectrum disorder and ADHD; motor developmental delay was based upon the physical therapist’s report and if available on a score below the fifth centile in the Movement Assessment Battery for Children; cognitive developmental delay was defined as an intelligence quotient less than or equal to 70 if this was tested, or it was based on a diagnosis by a medical specialist; speech and language development were assessed by the speech therapist or speech and hearing centre. Additionally, the severity of the LTI was assessed by accumulating the number of domains affected and indicated as the presence of LTI in two or more domains. Since in this cohort maternal seroimmunity to CMV before birth was unknown, it was assumed that cCMV infection could have resulted from either maternal primary or secondary infection. Due to the retrospective design of the study, there was no standardized clinical and laboratory assessment performed at birth. Therefore, we cannot exclude the possibility that we might have misclassified some newborns without clinically apparent disease or with mild and transient symptoms in the asymptomatic group. However, because of the Dutch child health care system, the chance of having missed major signs or symptoms can be considered negligible [23, 24].

For the study presented in this article, DBS were selected based on the clinical outcome of the infants, with a total of 6 CMV-negative without any clinical signs, 6 CMV-positive with LTI and 6 CMV-positive without LTI. This study was approved by the Medical Ethics Committee of the Leiden University Medical Center, and all the parents of the children included have given written informed consent for the use of clinical data and DBS.

### DNA extraction from DBS and qPCR of CMV

After a first initial CMV PCR screening performed at the National Institute for Public Health and the Environment (RIVM), a second confirmatory PCR was performed at the Leiden University Medical Center (LUMC) [23]. For this purpose, DNA was extracted from DBS by using the QIAamp DNA minikit according to the previously described protocol [25]. For each test, one full DBS was punched by using an automated DBS puncher (1296–071, Perkin Elmer-Wallac, Zaventem, Belgium). CMV DNA amplification of a 126-bp fragment from the immediate-early antigen region was performed using an internally controlled quantitative real-time PCR, as described previously [26, 27], on the CFX96 Real-Time PCR Detection System (BioRad, Veenendaal, The Netherlands). The PCR was performed in triplicate, and the CMV viral load was expressed in IU/ml.

### RNA extraction from DBS

One full DBS was punched using an automated DBS puncher (1296–071, Perkin Elmer-Wallac, Zaventem, Belgium). RNA was extracted from DBS by using the NucleoSpin miRNA kit (Macherey-Negel, Duren, Germany), according to the manufacturer’s instructions with a minor modification. This included pre-incubating the DBS with 300 μl of lysis buffer ML for 30 min at 37°C with agitation (1000 rpm) [28]. The supernatant was transferred to the NucleoSpin Filter, and the procedure was carried out according to the manufacturer’s instruction. Small and large RNAs were purified in one fraction, without separation of small RNAs, and a DNase treatment was used to reduce DNA contamination. The RNA was eluted in 50 μl of RNase-free H_2_O, and RNA integrity was assessed using the RNA Nano 6000 Assay Kit on the Bioanalyzer 2100 system (Agilent Technologies, CA, USA). The RNA concentration was measured using a Qubit 2.0 flurometer (Life Technologies, CA, USA).

### Library preparation and sequencing

An average amount of 185 ng of RNA was used as input material for library preparation. Sequencing libraries were generated using the TruSeq Stranded Total RNA Sample preparation kit for Illumina (Illumina, Inc., San Diego, CA, USA) following the manufacturer’s recommendations, and index codes were added to attribute sequences to each sample. Briefly, rRNA was depleted from total RNA using rRNA removal magnetic beads (RRB). The remaining RNA was purified using RNAClean XP magnetic beads. As the RNA samples from DBS were already fragmented, the fragmentation step was skipped in order to avoid over-fragmentation. First strand cDNA was synthetized using random hexamer primers and SuperScript II reverse transcriptase. Second strand synthesis was performed using the polymerase provided with the kit. After adenylation of the 3’ end of the blunt-ended DNA fragments, the RNA index adapters were ligated, and PCR was carried out using the PCR master mix and primer cocktail provided by Illumina to amplify the DNA in the library that had adapter molecules on both ends. Library quality was assessed using the DNA 1000 Assay kit for the Agilent Bioanalyzer 2100 system (Agilent Technologies, CA, USA), and the DNA amount was measured using a Qubit 2.0 flurometer. Clustering of the index-coded samples was performed using the Illumina TruSeq PE Cluster Kit v3 (cBot-HS) according to the manufacturer’s instructions. After cluster generation, the libraries were sequenced on the Illumina HiSeq 2000 platform (6 samples per lane), and 76 base paired-end reads were generated for the first batch (n = 6, 2 of each group) and 50 base paired-end reads for the second batch (n = 12, 4 of each group). All 76 base reads were trimmed to 50 bases to allow for uniform subsequent analysis across all samples, and the batch effect was accounted for in downstream analysis. Due to lack of resources it was not possible to sequence the whole cohort.

### Read mapping to the reference genome

Sequence files were generated in FASTQ format, and all RNA sequence files were processed using the BIOPET Gentrap pipeline version 0.7 developed at the LUMC (http://biopet-docs.readthedocs.io/en/latest/releasenotes/release_notes_0.7.0/). The BIOPET Gentrap pipeline consists of FASTQ pre-processing (including quality control, quality trimming and adapter clipping), RNA-seq alignment, read and base quantification. FastQC version 0.11.2 was used for raw read quality control. Low quality read trimming was done using sickle version 1.33 with default settings. Cutadapt version 1.9.1 with default settings was used for adapter clipping based on the detected adapter sequences by FastQC toolkit. RNA-seq reads were aligned against human reference genome GRCh38 using RNA-seq aligner GSNAP version 2014-12-23 with settings "—npaths 1—quiet-if-excessive". Ensembl human genome annotation version 87 was used for raw read counting. The gene read quantification step was performed using htseq-count version 0.6.1p1 with the setting "—stranded = reverse".

### Differential expression analysis: Individual genes

We identified significant gene expression differences between congenitally infected children (n = 12) and controls (n = 6), as well as between congenitally infected children that developed LTI (n = 6) and congenitally infected children that did not develop LTI (n = 6). Moreover, we also assessed gene expression differences in relation to logarithm of CMV viral load treated as continuous variable. Genes with low fragment counts were removed by requiring at least 2 fragments per million of aligned fragments to be observed in at least 2 samples. Library size normalization factors were obtained with the trimmed mean of M-values (TMM) method [29]. Linear modelling using Bioconductor/R package ‘limma’ [30] was performed on read counts transformed to log-CPM values. Observational-level weights obtained from the voom function were used to model mean-variance relationship. All three analyses were corrected for the batch effect in the design matrix. Multiple testing correction using false discovery rate control of Benjamini and Hochberg was performed at the threshold of 0.05.

### Differential expression analysis: Pathways

The Bioconductor/R package ‘global test’ designed by J. Goeman was used to evaluated differences in expression profiles of gene sets between the different groups [31]. These were a group of congenitally infected children (n = 12) and a group of controls (n = 6). Within the group of congenitally infected children, those that developed LTI (n = 6) and those that did not develop LTI (n = 6). An additional analysis was performed to find gene set expression profiles dependent on CMV viral load as continuous variable. This method has been shown to have more power to detect gene sets with small effect size [29, 32, 33]. We selected a limited number of candidate gene sets (pathways) for use in the global test, before inspecting the data using the QuickGO browser [34]. The pathways were selected based on their putative role in the etiology of the disease. An additional selection criterion was the specimen, i.e. DBS, which derives from whole blood and therefore mainly reflects the neonatal immune system. These pathways were T-, B-, and NK-cell activation, innate immune response, and inflammatory response with its regulation. Each pathway contained from 17 to 435 genes. This analysis was performed on the voom-transformed data. Due to the exploratory nature of this study, and to the limited number of selected pathways, no multiple testing correction was applied.

Finally, an additional immune pathway that has emerged as one of the possible players in limiting the immune response during cCMV is the T cell exhaustion [7]. However, this does not exist yet as a pathway in the QuickGo browser. Therefore, based on the transcriptional definition of exhaustion previously described [7, 35], and on our available data, a set of exhaustion genes was selected. An independent sample t-test was used to evaluate the difference in the square root of the reads per million (RPM) between the different categories. CMV+ vs CMV-, CMV+ without LTI vs CMV+ with LTI, CMV+ low load vs CMV+ high load. In the latter, the infected group was split in two according to the median log2 viral load measured in DBS which was 10.2, namely low (< 10.2) and high (≥ 10.2) viral load groups. However, p-values were not reported because this analysis had the sole purpose of illustrating trends.

## Results

### Study population and clinical data

The clinical data of the congenitally infected children included in this study, as well as of the non-infected controls, are listed in Table 1. A total of 12 children with cCMV, and 6 without cCMV, were included in order to assess the gene expression profile in relation to cCMV. Additionally, the 12 children with cCMV were selected in order to assess differences in gene expression in relation to LTI development. For this purpose, 6 infected children were selected, who did not have any symptoms at birth nor LTI at six years of age, whereas the other 6 had LTI in one or more of the following domains of impairment: neurological, motor, cognitive and speech/language (Table 1). Five children out of those who developed LTI also had symptoms at birth. Importantly, none of the children in the control group had symptoms at birth nor developed LTI. Given the diversity of the specific symptoms at birth and impairments at the age of six, the subjects were selected in order to have a similar proportion of male and female across the groups. In this way, the influence of gender in the gene expression analysis was limited.

**Table 1:** Study population and clinical outcome.

	cCMV with LTI^1^	cCMV no LTI^2^	No cCMV^3^
	n = 6	n = 6	n = 6
**Gender**			
Male	4	3	3
Female	2	3	3
**Gestational age (weeks)**^4^	39 (36–40)	40 (37–41)	41 (37–41)
**Birth weight (g)**^4^	3040 (1890–4040)	3340 (2760–4240)	3298 (3070–4360)
**CMV viral load**^5^	3.1 (2.43–4.97)	3.1 (2.18–4.30)	-
**Long term impairment**			
Hearing impairment^6^	0	0	0
Visual impairment^7^	0	0	0
Neurological impairment^8^	3	0	0
Motor impairment^9^	6	0	0
Cognitive impairment^10^	4	0	0
Speech/language problem^11^	4	0	0
**More than one impairment**^12^	5	0	0

1 Congenitally infected children that develop LTI, 5 out of 6 had symptoms at birth including prematurity (n = 1), dysmaturity (n = 1), microcephaly (n = 3)

2 Congenitally infected children that did not develop LTI, none of them had symptoms at birth

3 Non-infected controls, none of them had symptoms at birth nor LTI

4 Values are medians with minimum and maximum

5 CMV viral load measured on DBS, values are log (IU/ml) medians with minimum and maximum

6 Sensorineural hearing loss ≥ 40 decibels

7 Optic nerve atrophy or cortical visual impairment

8 Cerebral palsy (n = 1), epilepsy (n = 1), microcephaly (n = 1), autism (n = 2), ADHD (n = 1)

9 Motor impairment (fine, gross or balance) based on test or diagnosis or sensory processing disorder or developmental coordination disorder (n = 6)

10 Cognitive impairment based on test or diagnosis (n = 4)

11 Language impairment based on test or diagnosis, speech-impairment, oral motor skill difficulties or auditory processing disorder (n = 4)

12 Impairment in two or more domains of impairment: hearing, visual, neurologic, motor, cognitive, and speech-language.

### Library preparation and sequencing statistics

The average number of RNA-seq read pairs per sample was 38.5 million ± 4.8 million, with 38.9 million ± 5.4 million for the CMV- samples and 38.4 million ± 4.7 million for the CMV+ samples. Within the CMV+ samples, those without LTI generated 37.6 million ± 5.9 million paired-end reads, and those with LTI generated 39.1 million ± 3.6 million paired-end reads. The mean RNA fragment size was 285 ± 8 bp, and the mean DNA fragment size was 165 ± 8 bp. On average, 92.25% of bases exceeded Q30. The detailed information per sample is shown in Table 2.

**Table 2:** RNA-seq data per individual.

ID^1^	cCMV^2^	Gender^3^	LTI^4^	Input RNA (ng)^5^	RNA fragment size (bp)	DNA fragment size (bp)	Total number of read pairs^6^	Total bases^7^	Raw bases Q10+^8^	Raw bases Q20+^9^	Raw bases Q30+^10^
1	CMV-	m	no	160	274	154	36559606	3655960600	3623399872 (99.1%)	3582227412 (98.0%)	3398394726 (93.0%)
2	CMV-	f	no	200	286	166	33157384	3315738400	3286143611 (99.1%)	3247394425 (97.9%)	3076116879 (92.8%)
3	CMV+	f	no	200	278	158	29540831	2954083100	2927528373 (99.1%)	2892987291 (97.9%)	2740849118 (92.8%)
4	CMV+	f	no	200	282	162	31300956	3130095600	3102402121 (99.1%)	3066377738 (98.0%)	2905730026 (92.8%)
5	CMV+	f	yes	120	281	161	33323826	3332382600	3302791747 (99.1%)	3265370156 (98.0%)	3096491305 (92.9%)
6	CMV+	m	yes	200	282	162	39311864	3931186400	3897307546 (99.1%)	3853368879 (98.0%)	3657532714 (93.0%)
7	CMV-	f	no	200	285	165	45571592	4557159200	4499488134 (98.7%)	4432680661 (97.3%)	4219189147 (92.6%)
8	CMV+	f	yes	200	278	158	42119123	4211912300	4158027595 (98.7%)	4097388499 (97.3%)	3904204865 (92.7%)
9	CMV+	m	no	140	286	166	43750889	4375088900	4319612015 (98.7%)	4258366454 (97.3%)	4066273713 (92.9%)
10	CMV-	f	no	200	282	162	35039051	3503905100	3463528125 (98.8%)	3416670543 (97.5%)	3265849570 (93.2%)
11	CMV+	m	yes	200	289	169	40100801	4010080100	3951127179 (98.5%)	3878646166 (96.7%)	3587779279 (89.5%)
12	CMV+	m	no	200	298	178	41322383	4132238300	4081237277 (98.8%)	4023236869 (97.4%)	3838652400 (92.9%)
13	CMV-	m	no	200	287	167	45568773	4556877300	4486847437 (98.5%)	4416030829 (96.9%)	4204748423 (92.3%)
14	CMV+	m	yes	200	289	169	36627608	3662760800	3618784465 (98.8%)	3566791324 (97.4%)	3397972122 (92.8%)
15	CMV+	f	no	200	302	182	41894051	4189405100	4140976514 (98.8%)	4084677610 (97.5%)	3904733403 (93.2%)
16	CMV-	m	no	200	271	151	37242565	3724256500	3638553568 (97.7%)	3565521893 (95.7%)	3317637210 (89.1%)
17	CMV+	m	yes	140	290	170	43235245	4323524500	4228806228 (97.8%)	4149633476 (96.0%)	3947504971 (91.3%)
18	CMV+	m	no	170	282	162	38075664	3807566400	3753496088 (98.6%)	3696000143 (97.1%)	3467378795 (91.1%)

1 ID child identification number

2 cCMV, congenital Cytomegalovirus infection; CMV+, congenitally infected children; CMV-, non-infected controls

3 f, female; m male

4 LTI, long-term impairment at 6 years of age

5 Input RNA (ng) the amount of RNA used as input material for library preparation;6 Total number of paired-end reads, total number of paired-end reads that passed Illumina filter generated per sample

7 Total bases, total number of bases generated per sample

8 Raw bases Q10+, base calls with quality Q-scores of Q10+ (Q10 or higher) have an error probability of 0.1 (1 in 10) or less

9 Raw bases Q20+, base calls with Q20+ have an error probability of 0.01 (1 in 100) or less

10 Raw bases Q30+, base calls with Q30+ have an error probability of 0.001 (1 in 1,000) or less.

### Differential expression: Individual genes

Next, we determined whether any other gene could be associated with cCMV, LTI development at 6 years of age or CMV viral load. After low count features removal, ~25% of counts aligned on features and 18360 different genes were used in gene expression analysis. The R package LIMMA was used for the assessment of differential expression of individual genes between congenitally infected children (n = 12) and non-infected controls (n = 6). No statistically significant differences in gene expression were observed between the groups. We next assessed gene expression differences in relation to cCMV clinical outcome by comparing congenitally infected children that developed LTI at six years of age (n = 6) to congenitally infected children that did not develop LTI (n = 6). This analysis did not reveal any statistically significant differences between the groups. Finally, the differences in gene expression were assessed in relation to the logarithm of CMV viral load as continuous variable, and no statistically significant differences were observed.

### Differential expression: Pathways

**Fig 1:**
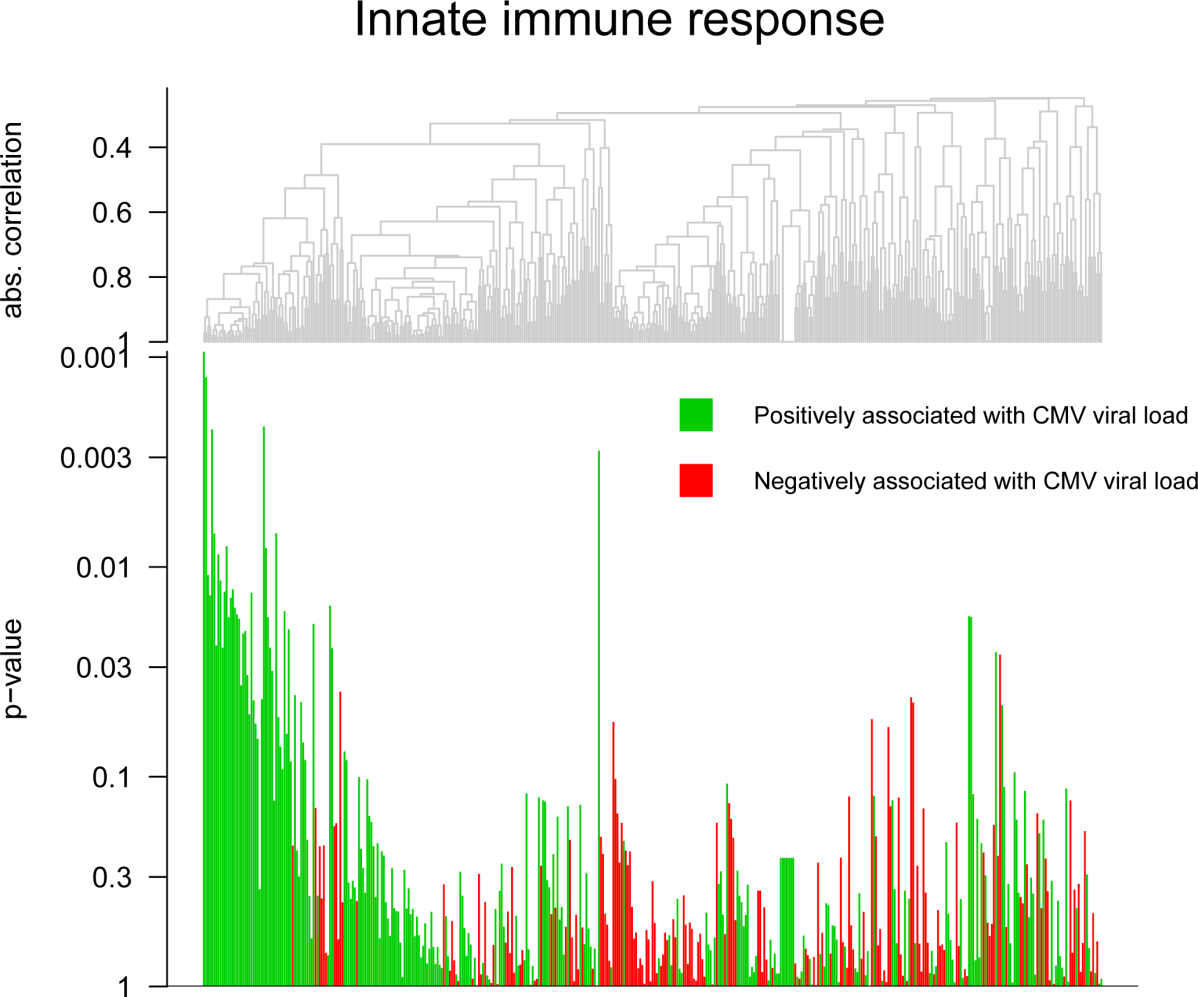
Global test: Innate immune response. Innate immune response in relation to CMV viral load as continuous variable measured on DBS, p = 0.046. The gene names of x-axes are provided in supplementary S1 Table.

**Fig 2:**
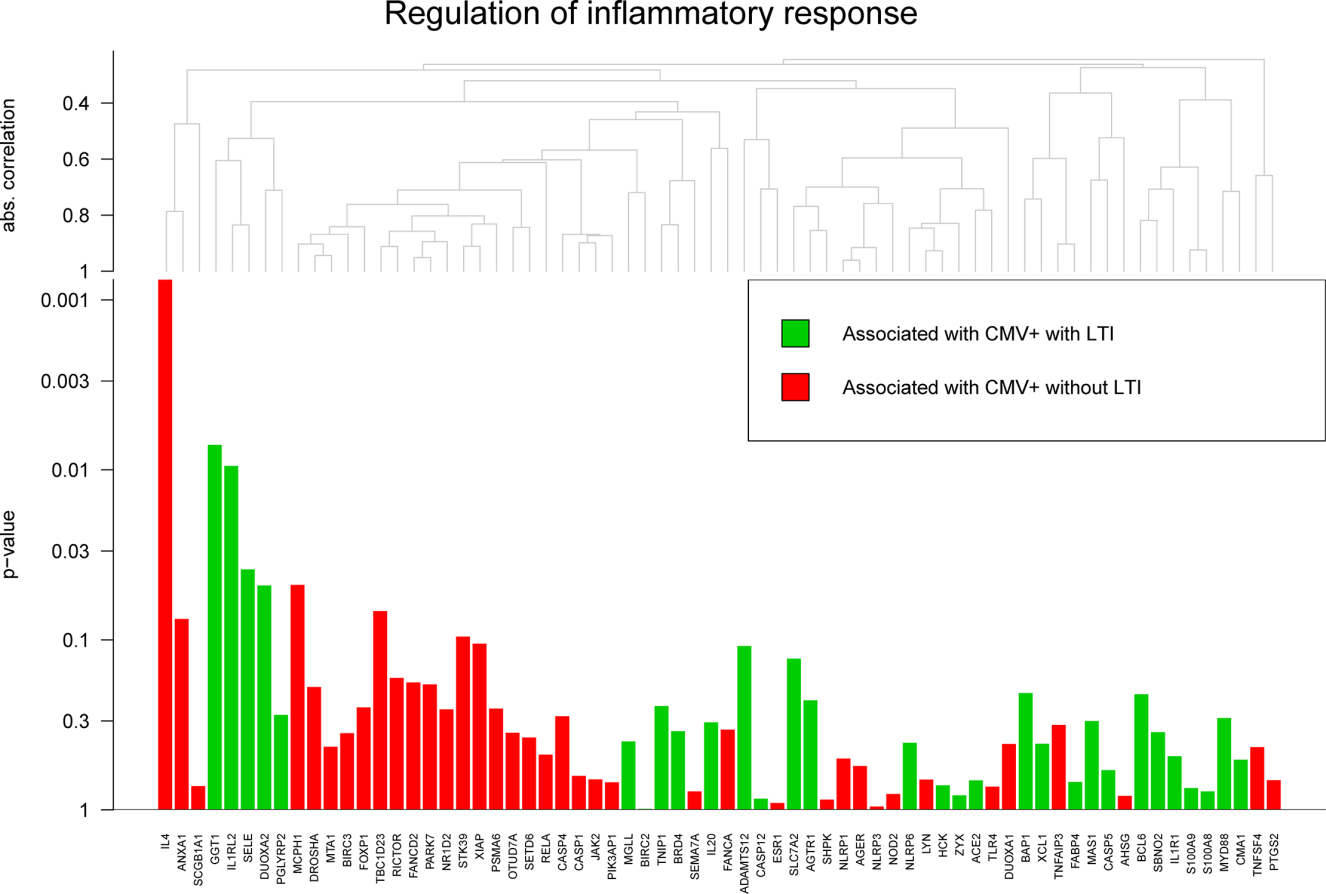
Global test: Regulation of inflammatory response. Regulation of inflammatory response in congenitally infected children that developed LTI at 6 years of age (n = 6) and in congenitally children that did not develop LTI (n = 6), p = 0.077.

In order to evaluate whether different biological mechanisms may underlie different clinical outcomes, a global test was performed on manually pre-selected pathways based on their putative role in the etiology of cCMV disease. The selected pathways for T-, B-, and NK-cell activation, innate immune response, and inflammatory response were assessed in relation to cCMV, LTI development at 6 years of age and CMV viral load. The results are shown in Table 3. This analysis revealed trend significant results in relation to CMV viral load and LTI development. In particular, the innate immune response (p = 0.046, Fig 1) and the NK-cell activation (p = 0.086) may be associated to CMV viral load; whereas the regulation of inflammatory response (p = 0.077, Fig 2) to LTI development. In all cases, a small number of genes appeared to be responsible for these trends. Several antiviral genes were positively associated with CMV viral load, i.e. ISG15 and RSAD2, whereas the anti-inflammatory cytokine IL-4 was associated with the congenitally infected children that did not develop LTI.

**Table 3:** Global test analysis.

Pathways	cCMV^1^	LTI^2^	CMV viral load^3^
	p-values		
**Innate immune response (435 genes)**	0.706^4^	0.432	0.046
**T cell activation (51 genes)**	0.375	0.203	0.195
**B cell activation (30 genes)**	0.367	0.125	0.254
**NK cell activation (17 genes)**	0.717	0.499	0.086
**Inflammatory response (381 genes)**	0.725	0.341	0.232
**Regulation of inflammatory response (68 genes)**	0.577	0.077	0.567
**Negative regulation of inflammatory response (78 genes)**	0.633	0.339	0.133
**Positive regulation of inflammatory response (74 genes)**	0.444	0.652	0.791

^1^ Gene sets expression differences between CMV- (n = 6) and CMV+ (n = 12)

^2^ Gene sets expression differences between congenitally infected children with LTI (n = 6) and without LTI (n = 6)

^3^ Gene sets expression differences according to CMV viral load (continuous variable).

**Fig 3:**
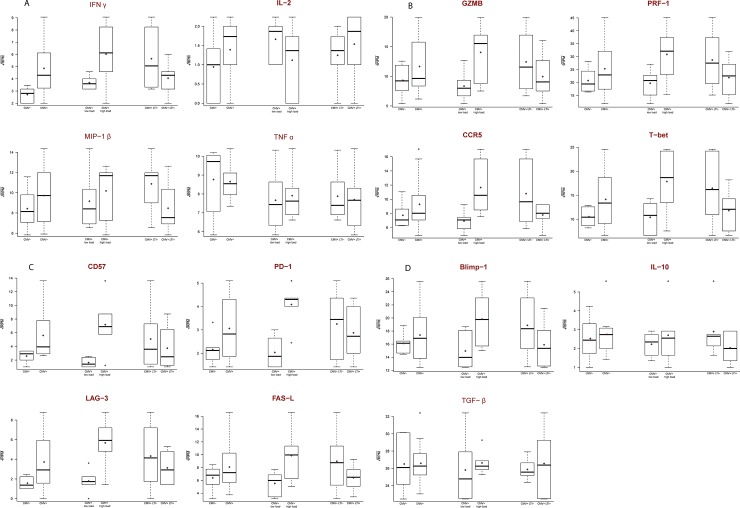
**A-D. T cell exhaustion.** T cell markers identifying the exhaustion phenotype in relation to cCMV, CMV viral load and LTI development at 6 years of age. *CMV-*, non-infected controls (n = 6); *CMV+*, congenitally infected children (n = 12); *CMV+ low load*, congenitally infected children with log2 CMV viral load below the median measured in DBS which was 10.2 (n = 6); *CMV+ high load*, congenitally infected children with log2 CMV viral load equal to or above the median measured in DBS (n = 6); *CMV+ LTI-*, congenitally infected children that did not develop LTI (n = 6); *CMV+ LTI+*, congenitally infected children that developed LTI (n = 6). Boxplot: *bold line*, median of square root of RPM; *red dot*, mean of square root of RPM.

Finally, as previously shown by others, one of the possible mechanisms limiting the T cell response to CMV during early life is considered to be T cell exhaustion [7]. Therefore, we wondered whether the same phenomenon could be observed in our cohort when comparing the CMV- group (n = 6) to the CMV+ group (n = 12). Additionally, this pathway was assessed in relation to CMV viral load and development of LTI at 6 years of age. For this purpose, based on the transcriptional definition of exhaustion previously described [7, 35], as well as on our available data, a set of genes was selected and reported in Table 4. Of these genes, the RPM were reported for each comparison in order to observe the trend to be further explored. A trend of increased expression of differentiation markers, mainly CD57 and transcription factor T-bet, and of increased effector markers, primarily IFN-γ and granzyme, was observed in the CMV+ group compared to the CMV- group (Fig 3A–3D). Furthermore, a trend of increased expression of inhibitory markers, mainly PD-1 and LAG-3, was observed in the CMV+ group (Fig 3A–3D). Next, the CMV+ group was split in two groups according to the median log2 viral load measured in DBS which was 10.2, namely low and high viral load groups. Comparing the group with high viral load to the one with low viral load, the aforementioned observed trends relative to differentiation, effector and inhibitory markers were more pronounced than when comparing CMV+ to CMV-. Finally, when comparing the cCMV+ group that developed LTI to those who did not, no striking trends were observed (Fig 3A–3D).

**Table 4:** T cell markers.

T cell markers
**Differentiation and effectors**^1^
IFNγ
IL-2
MIP-1β
TNF-α
Granzyme B
Perforin 1
CCR5
CD57
**Transcription factors**^2^
T-bet
Blimp-1
**Inhibitory receptors**
PD-1
LAG-3
FAS-L
**Inhibitory cytokines**
IL-10
TGF-β

1 Markers defining a differentiation phenotype that leads to a functional response

2 Key transcription factors for T cell differentiation and exhaustion

## Discussion

This study aimed to evaluate whether transcriptome analysis by next generation RNA sequencing on DBS derived from a retrospective nation-wide cohort of children with cCMV and controls is feasible, and whether useful insights could be obtained on the etiology of different cCMV outcomes. This would allow the identification of potential biomarkers for long-term outcome, which could provide the means to introduce the long-debated newborn screening program for cCMV in DBS [22]. Indeed, this would define subgroups of children benefitting from clinical, audiological follow-up, and possibly antiviral treatment.

The global test for differential expression of gene sets revealed, although only with trend significant results, an important feature of cCMV in relation to whole blood transcriptome, i.e. CMV viral load is the main factor to influence the pre-selected immune pathways, whereas CMV disease seems to be secondary. In our study, numerous antiviral genes were positively associated with CMV viral load, suggesting the involvement of the innate immune system in response to cCMV in the newborns, in particular with higher viral loads. The fact that no striking differences were observed when comparing CMV+ to CMV-, suggests that the high viral load is the main initiator of this expression pattern. Therefore, the presence of neonates with low viral load in the CMV+ group may have diluted the differences between CMV+ and CMV-. Congenitally infected children excrete CMV for several years after birth, whereas in adults this lasts only several months [36, 37], suggesting a deficient cell-mediated immune response in early life [38]. Therefore, it is tempting to speculate that the activation of the innate immunity in the fetus may have an important role in controlling cCMV, however this is difficult to determine. One of the possible mechanism for this limited T cell response to CMV during early life is considered to be T cell exhaustion [7]. In our cohort, also the exhaustion pathway was more pronounced in the high viral load group compared to the low viral load group, with PD-1 being the marker influenced the most, as previously shown [7]. Therefore, also in this case the difference in exhaustion pathway between CMV+ and CMV- could have been diluted because of the presence of low viral load individuals in the CMV+ group. However, the exhaustion pathway analysis needs further confirmation as we only reported expression trends. T cell exhaustion is characterized by loss of T cell functions, and is induced by persistent infections [7, 35]. Primary CMV infection induces functional T cell exhaustion in both adults and fetuses, though considerably more pronounced in the latter. As this phenomenon is associated with prolonged exposure with higher viral loads, the high viral loads reported in fetuses may be the cause of this effect [39–41]. The exhaustion may contribute to the prolonged CMV viral excretion in the children [7]. The influence of viral load in the immune responses has been shown before, both in humans and in the murine models of CMV infection. Here, the degree of CMV-specific memory CD8 T cells accumulation, as well as the phenotypic T cell profile, was influenced by the viral load [42, 43]. However, the role of CMV viral load in the clinical outcome still remains controversial. Some studies have correlated CMV viral load, measured in blood, with clinical outcome [44, 45], whereas others have not [46–48]. The neonatal viral load may differ depending on the trimester of vertical transmission, or whether it was a primary maternal infection. Indeed, earlier infections may lead to a more extensive cCMV. However, in our cohort this is impossible to establish [49]. Additionally, CMV viral load in whole blood may not correlate to CMV loads in other neonatal compartments, and therefore may not fully reflect viral replication in all affected organs and tissues.

The molecular mechanisms of LTI development are largely unknown, though the late-onset hearing loss is believed to be the result of a chronic productive infection throughout childhood [50, 51]. In this context, a long-term dysfunctional immune response seems plausible, although it cannot be excluded that such dysfunction leads to a parallel uncontrolled inflammation that contributes to tissue damage. In studies of characterization of tissue damage in fetuses of 20–21 weeks of gestation with cCMV, an association between the degree of tissue damage in the brain, as well as in the inner ear, with viral load, inflammatory response and placental functionality was shown [52, 53]. A dysfunctional immune response that leads to uncontrolled viral replication, and immune-mediated damage was suggested. Therefore, a similar pathogenesis may be assumed when such infection becomes chronic. The exhaustion pathway that was found in congenitally infected children, especially those with higher CMV viral load, did not seem to correlate to clinical outcome at 6 years of age. This suggests that other mechanisms are involved in the long-term immune dysfunction. In our cohort, when comparing congenitally infected children that developed LTI to those infected who did not, a role for the regulation of inflammatory responses seemed to partially contribute. Anti-inflammatory markers, such as the cytokine IL-4, were associated with congenitally infected children that did not develop LTI. The success of an immune response is the result of a balance between effector and regulatory mechanisms, therefore, the potential protective effect of IL-4 in those infected children that did not develop LTI may lie in its anti-inflammatory property. Interestingly, in a cohort of healthy CMV infected individuals, the CD4 T-cell response associated with a protective immunity involved cytokine production of IFN, and/or IL-17, in association with IL-4 [54]. Similarly to IL-10, IL-4 has been shown to possess the capacity of down-regulating the production of pro-inflammatory mediators by microglia, both in humans and in mice [55–57], and its neuroprotective effect was associated with downregulation of brain inflammation in mice [58]. When studying the regulation of the inflammatory response in children with cCMV and compare the group with LTI to that without LTI, we have to be aware that there may be other perinatal factors influencing the inflammatory pattern in DBS. Although we cannot fully exclude a role for non-cCMV related perinatal factors, there was no bacterial amniotic infection or neonatal sepsis in all children included in this study.

Several reasons may have contributed to the fact that we did not find a strong impact of cCMV on whole blood transcriptomes from DBS. First of all, one of the groups of congenitally infected children did not have symptoms at birth nor LTI, which is the case in most children with cCMV, and the clinical signs of symptoms associated with LTI are very diverse. Second of all, in our cohort, the fetal infection may have been the result of a primary or secondary CMV infection in the mother, and may have taken place at any time during pregnancy, especially in the asymptomatic children. Third of all, the small sample size of the groups may have led to a lack of power both in the gene expression analysis of individual genes, as well as in the pathway analysis. Lastly, the RNA degradation on these specimens, due to e.g. ribonucleases, pH, humidity or UV light, may have contributed to the lack of significant differences among the sample groups. The degradation of RNA from dried stains has been extensively studied in forensic studies for obvious reasons, and several RNAs have been extracted from numerous conditions [59–64]. From these studies, determinants for RNA stability appeared to be the specimen the RNA is extracted from, and the specific RNA molecule analyzed. In the former, the detection limit of blood-specific RNA has been shown to be lower than for other specimens [21]. In the latter, some RNAs can be more stable in dried stains than others [21]. Secreted RNAs, e.g. in fresh saliva, may be more susceptible to fast degradation by extracellular RNases, and therefore are not to be expected on dried stains [20]. Importantly, for those RNAs detected on dried blood stored at room temperature, only few genes have been demonstrated to be differentially expressed during time [20]. Therefore, we assumed that those markers detected on DBS in our study were less prone to degradation, and relatively stable for long periods of time. Furthermore, the influence of RNA contamination in the downstream analysis, e.g. from skin cells or external microorganisms, may be considered negligible as the most abundant RNAs species come from the host whole blood [65]. Despite the fact that enough data were generated in our study for the downstream analysis, with comparable cDNA fragment size as shown in forensic studies [21, 59–64], we cannot exclude that fresh material may have revealed differences in expression patterns that we could not pick up.

Furthermore, due to the retrospective nature of the study, cCMV diagnosis was performed by performing PCR of viral DNA on DBS, which in comparison with PCR on urine or saliva has been associated with limited and variable sensitivity [66]. Therefore, a negative CMV PCR on DBS does not fully exclude cCMV. However, it is important to note that with the relatively high sensitivity of our CMV PCR on DBS (estimated > 85%), high specificity (> 99.9%) and the cCMV birth prevalence of 0.5%, the chance of a CMV false-negative result is 1/1000 [23]. Therefore, it is very unlikely that a cCMV positive child ended up in our cCMV negative control group.

To the best of our knowledge, this is the first exploratory study assessing the feasibility of transcriptome sequencing using RNA isolated from archived neonatal DBS of children with cCMV, and non-infected controls, in relation to long-term outcome. Despite the lack of statistical power to detect individual gene expression differences, the pathway analysis suggested a potential differential gene expression in relation to CMV viral load and LTI. Therefore, this study represents a first step in unraveling the pathogenesis of cCMV, and in identifying prognostic markers for cCMV long-term outcome.

## Supporting information

S1 Table Gene names. Gene names of x-axis of Fig 1 concerning the innate immune response in relation to CMV viral load as continuous variable.
(XLSX)

S2 Table Read counts per gene. (XLSX)

## Abbreviations

cCMV: congenital Cytomegalovirus infection
DBS: dried blood spots
LTI: long-term impairments
CMV+: children with Ccmv
CMV-: children without cCMV
RPM: reads per million
